# Non-verbal sensorimotor timing deficits in children and adolescents who stutter

**DOI:** 10.3389/fpsyg.2015.00847

**Published:** 2015-07-06

**Authors:** Simone Falk, Thilo Müller, Simone Dalla Bella

**Affiliations:** ^1^CNRS, Laboratoire Parole et Langage, UMR 7309, Aix-Marseille UniversityAix-en-Provence, France; ^2^Institut für Deutsche Philologie, Ludwig Maximilians UniversityMunich, Germany; ^3^Neurology (Stuttering Therapy), LVR HospitalBonn, Germany; ^4^Movement to Health Laboratory, EuroMov, University of MontpellierMontpellier, France; ^5^Institut Universitaire de FranceParis, France; ^6^Department of Cognitive Psychology, Wyższa Szkoła Finansów i ZarządzaniaWarsaw, Poland

**Keywords:** developmental stuttering, predictive timing, sensorimotor synchronization, children, auditory–motor coupling, rhythm

## Abstract

There is growing evidence that motor and speech disorders co-occur during development. In the present study, we investigated whether stuttering, a developmental speech disorder, is associated with a predictive timing deficit in childhood and adolescence. By testing sensorimotor synchronization abilities, we aimed to assess whether predictive timing is dysfunctional in young participants who stutter (8–16 years). Twenty German children and adolescents who stutter and 43 non-stuttering participants matched for age and musical training were tested on their ability to synchronize their finger taps with periodic tone sequences and with a musical beat. Forty percent of children and 90% of adolescents who stutter displayed poor synchronization with both metronome and musical stimuli, falling below 2.5% of the estimated population based on the performance of the group without the disorder. Synchronization deficits were characterized by either lower synchronization accuracy or lower consistency or both. Lower accuracy resulted in an over-anticipation of the pacing event in participants who stutter. Moreover, individual profiles revealed that lower consistency was typical of participants that were severely stuttering. These findings support the idea that malfunctioning predictive timing during auditory–motor coupling plays a role in stuttering in children and adolescents.

## Introduction

There is growing evidence that speech and non-verbal motor functions are closely interrelated during development. In the first months of life, infants produce their first language-specific vocalizations (“canonical babbling”) at the same moment when they begin to master rhythmic limb movements (Kent and Bauer, 1985; Davis and MacNeilage, 1995; Ejiri and Masataka, 2001). Recent research has also shown that developmental speech and language disorders often are accompanied by deficits in non-verbal fine motor functions (e.g., Owen and McKinlay, 1997; Iverson and Braddock, 2011). For example, children with speech sound disorders or dyslexia have more difficulties than normally developing children with tasks demanding fine-grained temporal motor adaptation such as when synchronizing to rhythmic sounds or music or when imitating a rhythm via a motor manual response (Peter and Stoel-Gammon, 2008; Thomson and Goswami, 2008; Tierney and Kraus, 2013; Flaugnacco et al., 2014; Redle et al., 2014). These findings suggest that developmental deficits in motor and speech functions may emerge together because they stem from a common underlying deficit (Redle et al., 2014). In the present article, the possibility is examined for stuttering, a developmental speech disorder with a particular focus on timing mechanisms.

Stuttering is a disorder that is very disruptive in conversation. During speech production, persons who stutter suffer from frequent, involuntary disruptions in their forward flow of speech. These disruptions, either silent or audible, occur as blocks, as prolongations and repetitions of sublexical units such as segments, syllables, or part-words and are often associated with high effort or tension in the speaker’s production (e.g., Wingate, 1964; Starkweather, 1987). These disfluencies tend to increase in demanding and stressful situations and are experienced as a loss of control (Perkins, 1990). According to the WHO classification, stuttering is characterized as a major disruption of the rhythmic flow of speech (WHO, 2015). Despite the fact that persons who stutter know exactly what they want to say and do not suffer from problems in accessing words, they “find themselves unable to initiate or complete motor execution of words in a timely manner” (Brocklehurst, 2013, p. 291). Stuttering is a disorder that arises mostly at preschool age (3 or 4 years) and affects 5–9% of children during childhood and adolescence (Bloodstein and Bernstein Ratner, 2008; Yairi and Ambrose, 2013). In approximately 1% of the population, stuttering persists into adulthood.

A variety of explanations have been proposed to account for stuttering phenomena (Ward, 2006; Howell, 2011; Guitar, 2014). Many researchers agree that, besides linguistic and psychological aspects, speech motor control plays a major role in the disorder (e.g., Zimmermann, 1980; Ludlow and Loucks, 2003; Civier et al., 2010; Namasivayam and van Lieshout, 2011). Within the motor system, timing mechanisms have been targeted as a potential source of deficits underlying stuttering (Cooper and Allen, 1977; Van Riper, 1982; Borden, 1983; Kent, 1984; Harrington, 1988; Boutsen et al., 2000; Max and Yudman, 2003; Olander et al., 2010; Etchell et al., 2014). This hypothesis is in keeping with the observation that speech production in persons who stutter is temporally more variable than in individuals without stuttering, notably, in the temporal dynamics of subglottal air pressure, in articulatory kinematics, in utterance length, vowel duration, or voice onset time (e.g., Cooper and Allen, 1977; Kleinow and Smith, 2000; Smith and Kleinow, 2000; Max and Gracco, 2005). Interestingly, timing deficits were also found in non-verbal motor tasks, in studies which were almost exclusively conducted with adult speakers. Increased motor execution times and delayed initiation in non-verbal oral as well as manual reactions and longer movement sequences were found in adults who stutter compared to non-stuttering adults (e.g., Borden, 1983; Hulstijn et al., 1992; Archibald and De Nil, 1999; Smits-Bandstra et al., 2006). Decreased accuracy was found during bimanual finger coordination tasks (Zelaznik et al., 1997), as well as increased variability in self-paced tapping (Cooper and Allen, 1977) and in joint synchronization of speech and tapping to tone sequences (Hulstijn et al., 1992). However, these findings were not always replicated in similar studies (Zelaznik et al., 1994; Max and Yudman, 2003; Neef et al., 2011). Inconsistent findings in adults may result from task and measurement factors. Indeed, it was more likely to uncover deficits when using more demanding tasks and when examining articulatory kinematics in addition to acoustic measurements in non-speech oral movements (e.g., Hulstijn et al., 1992). In sum, results showing temporal deficits in both verbal and non-verbal tasks are compatible with the idea that speech and non-verbal motor deficits may jointly emerge from a common malfunctioning timing mechanism in stuttering.

In keeping with the possibility of a timing disorder in stuttering, there is also evidence from functional and structural neuroimaging studies that persons who stutter differ from persons who do not stutter with regard to the timing system (see Etchell et al., 2014, for a review). Differences are found in adults at the level of the basal ganglia (e.g., Alm, 2004; Giraud et al., 2008; Chang and Zhu, 2013; Civier et al., 2013) and of the cerebellum (e.g., Brown et al., 2005) in a variety of speech production tasks. These regions play a crucial role in mediating the flow of information for temporal fine-tuning and coordination of motor responses as well as for sensorimotor integration (Wing, 2002; Zatorre et al., 2007; Kotz and Schwartze, 2010). In adults and adolescents who stutter, reduced connectivity was found in the three cerebellar peduncles (Connally et al., 2014) and hyperactivation of the cerebellar vermis, a region typically involved in motor control, was also reported (see Brown et al., 2005, for a review). Furthermore, Giraud et al. (2008) found a positive correlation between stuttering severity and the activation of the nucleus caudate and a negative correlation with activation in the left substantia nigra. A few other studies also point to differences between persons who do and do not stutter in the basal ganglia-thalamocortical circuit (Fox et al., 2000; Ingham et al., 2004; Watkins et al., 2008; Lu et al., 2010; Chang and Zhu, 2013). In the only study on young children (4–9 years), Chang and Zhu (2013) found attenuated activity and reduced connectivity in the basal ganglia-thalamocortical circuit and in the auditory–motor cortical loops in the left hemisphere, potentially associated with deficits in self-initiated timing of speech movement and auditory feedback integration in stuttering. Altered connectivity between the basal ganglia and the premotor area was found during speech planning in adults who stutter while the cerebellar-premotor circuit was more affected during speech production (Lu et al., 2010). At the cortical level, differences in further areas and connections related to the subcortical motor timing circuitry and important hubs for sensorimotor integration have been found in persons who stutter. For instance, the supplementary motor area (SMA; e.g., Casini and Vidal, 2011; Kotz and Schwartze, 2011) was characterized by lower amplitude of low-frequency fluctuations in a resting-state MRI study in adults who stutter compared to adults who do not stutter (Xuan et al., 2012). During speech production in persons who stutter, however, the SMA showed hyperactivation in other studies (see Brown et al., 2005). Another area of interest for timing that is often pointed out is the left ventral premotor cortex (e.g., Watkins et al., 2008; Civier et al., 2013). Functionally, this area is associated with articulatory planning and integration of motor actions with their sensory consequences (Wise et al., 1999; Kohler et al., 2002). Adults and adolescents who stutter displayed reduced integrity of white fiber tracts in this area (Watkins et al., 2008).

Altogether, there is converging behavioral and brain evidence that stuttering in adult speakers is associated with deficient temporal processing in both verbal and non-verbal domains. However, whether these deficits are also found during development, when stuttering is more prevalent, is still unclear. There is a paucity of studies on non-verbal timing mechanisms in children suffering from developmental stuttering. In the few non-verbal motor timing studies with children who stutter, Howell et al. (1997) and Olander et al. (2010) reported higher variability in synchronization–continuation tasks (Wing, 2002). In these tasks, children were asked to synchronize their hand or non-speech lip movements to an auditory or visual rhythmic stimulus and to continue these movements at the same pace after the end of the pacing stimulus. Olander et al. (2010) tested 17 children between 4 and 6 years of age. The children were asked to clap along with a metronome with an Inter-Onset-Interval (IOI) of 600 ms, and then to continue at the same pace after the end of the metronome. Fifty-nine percent of the children who stutter were more variable in interclap-interval rate in the continuation phase compared to age-matched peers. However, these findings have to be considered with caution, as they are not confirmed with a larger sample size^1^. Howell et al. (1997) reported higher variability in the continuation phase of non-speech lip movements in five children aged 9–10 years. Analyses in this study indicated that the variability was more likely to pertain to the planning and realization of motor actions than to central cognitive mechanisms, such as an internal biological clock or timekeeper (Wing and Kristofferson, 1973; Howell et al., 1997).

Malfunctioning timing mechanisms are likely to negatively affect speech production in individuals who stutter. Some models of stuttering posit that a major problem lies in deficient auditory–motor integration or forward modeling (e.g., Neilson and Neilson, 1987; Max et al., 2004). More specifically, Harrington (1988) hypothesized that mechanisms related to the prediction and integration of the moment of one’s own speech production with the moment of actual sensory feedback are malfunctioning in persons who stutter. In Harrington’s (1988) model it is hypothesized that persons who stutter expect the time of sensory feedback of their own productions to occur earlier than it actually does. Thereby, they would erroneously correct for the moment of their actual segmental production, thus sometimes leading to repetitions, prolongations or even blockades of the articulatory gestures. The idea of erroneous predictive timing in the case of stuttering of stuttering is attractive in light of the finding that external predictable temporal cues can considerably aid persons who stutter to speak fluently (e.g., Johnson and Rosen, 1937; Wingate, 1969; Andrews et al., 1982; Stager et al., 2003; Toyomura et al., 2011). For example, stuttering symptoms are reduced when speech production is paced by an isochronous tone sequence (i.e., in paced or metronomic speech) or by the speech of another person (e.g., in shadowed speech, choral reading) or choral singing. Interestingly, improved fluency induced by rhythmic cues is accompanied by a normalization of hyper- and hypo-activation in neural circuits mediating temporal processing and movement initiation, such as the basal ganglia, SMA, and the cerebellum (Fox et al., 1996; Stager et al., 2003; Toyomura et al., 2011). To account for these effects, Stager et al. (2003) proposed that fluency-evoking conditions aid persons who stutter to achieve better coupling of their auditory and motor systems.

In the present study, we tested the hypothesis that children and adolescents who stutter show deficits in non-verbal timing abilities, with a particular focus on predictive timing, which is tested using a sensorimotor synchronization task. Sensorimotor synchronization is ideally suited to test predictive timing as it involves coupling of fine motor movement with a predictable sound sequence (e.g., a metronome or music; Repp, 2005; Neef et al., 2011; Repp and Su, 2013). This task was used here to test timing abilities in young participants (8–16 years), from childhood to adolescence, as stuttering is more prevalent in this age group than in adults. An additional goal of the study was to examine individual profiles of young participants who stutter to shed light on the link between stuttering severity and non-verbal motor timing. Indeed, we expect a malfunctioning timing system (e.g., inaccurate predictive timing) to particularly characterize the performance of those individuals showing most severe stuttering. In previous studies, sensorimotor synchronization has proven to be particularly sensitive to individual differences in unimpaired and impaired individuals (e.g., patients with movement disorders, such as Parkinson’s disease, or individuals with beat deafness; Sowiński and Dalla Bella, 2013; Benoit et al., 2014). Notably, to our knowledge, this is the first study to examine sensorimotor synchronization in children and adolescents who stutter as the previous studies on motor timing skills in these populations focused on unpaced movement (i.e., the continuation phase in a synchronization–continuation task; Howell et al., 1997; Olander et al., 2010).

Participants’ synchronization abilities were assessed with a finger tapping task (Repp, 2005, 2006; Repp and Su, 2013). They tapped along with the rhythm of non-verbal auditory pacing stimuli such as isochronous tone sequences (i.e., a metronome). The difficulty of the task was varied by speeding up or slowing down the metronome and by varying the complexity of the pacing stimulus by using musical excerpts which have a complex rhythmic structure. A timing deficit in children and adolescents who stutter is expected in terms of lower synchronization accuracy, particularly indicative of predictive timing, as well as lower synchronization consistency (i.e., higher variability) as compared to children and adolescents who do no stutter. In addition, differences between the two groups are expected to be more visible when increasing task difficulty.

## Materials and Methods

### Participants

Sixty-three native German-speaking children (*M* = 10.3 years) and adolescents (*M* = 14.5 years) participated in the Experiment. Each age group consisted of participants with developmental stuttering (10 children, 3 females, 7 males, *M =* 9.9 years, *SD =* 0.99; 10 adolescents, 2 females, 8 males, *M* = 14.0 years, *SD* = 1.6) and a randomly selected age-matched control group (22 children, 8 females, 14 males, *M =* 10.5 years, *SD =* 0.5; 21 adolescents, 8 females, 13 males, *M* = 14.7 years, *SD* = 0.6). Participants who stutter were recruited and tested prior to a therapy course held in the surroundings of Munich in summer 2012 and 2013 (staerker-als-stottern.de). Participants had on average 2.4 years of musical training (range = 0–8.5 years, *SD* = 2.3). Neither experimental and control groups nor the age groups differed in terms of musical training. Stuttering severity was assessed with the Stuttering Severity Instrument (SSI-3, Riley, 1994) and the German FzS (‘Fragebogen zum Sprechen’, Cook et al., 2013), the latter being an assessment of the psychosocial impact of stuttering on the everyday life of the participants. SSI scores ranged from very mild to very severe stuttering as did the scores for the psychosocial impact (see **Table 1**). The study was carried out in conformity with ethical standards. Participants and their parents gave informed consent to participate in the study.

**Table 1:** Characteristics of participants who stutter including stuttering assessment [Stuttering Severity Instrument, (SSI-3) and Fragebogen zum Sprechen (FzS)].

Participant number	Age (years)	Gender	SSI-3	FzS	Musical training (years)
S01	15	Male	52^∗∗∗^	130^∗∗∗^	2
S02	9	Male	15^∗^	92^∗∗∗^	0
S03	15	Male	27^∗∗^	53^∗^	5
S04	11	Female	24.5^∗∗^	81^∗∗^	0
S05	15	Male	27.5^∗∗∗^	89^∗∗^	0
S06	13	Male	33^∗∗∗^	73^∗∗^	0
S07	9	Male	26.5^∗∗^	42^∗^	0
S08	10	Male	17^∗^	63^∗^	3
S09	11	Male	18^∗^	114^∗∗∗^	3
S10	12	Female	19^∗^	98^∗∗∗^	4
S11	16	Female	30.5^∗∗∗^	97^∗∗∗^	2
S12	12	Male	28^∗∗∗^	62^∗^	0
S13	10	Male	30^∗∗∗^	51^∗^	0
S14	10	Female	10^∗^	76^∗∗^	2
S15	15	Male	38^∗∗∗^	106^∗∗∗^	8
S16	12	Male	6^∗^	41^∗^	4
S17	11	Male	35^∗∗∗^	63^∗^	0
S18	15	Male	28^∗∗∗^	105^∗∗∗^	1,5
S19	8	Female	21^∗∗^	88^∗∗^	3
S20	10	Male	8^∗^	75^∗∗^	3

*Degree of stuttering severity: ^∗∗∗^severe, ^∗∗^moderate, ^∗^mild.*

### Stimuli and Procedure

Participants performed the sensorimotor synchronization tasks taken from the Battery for the Assessment of Auditory Sensorimotor and Timing Abilities (BAASTA – Benoit et al., 2014). They synchronized with three isochronous sequences of tones referred to as “metronome” and two musical excerpts. They tapped along with the tones or the beat of the music using the index finger of their dominant hand. The metronome sequences were presented at three different tempi and consisted of 60 tones (frequency = 1319 Hz) separated by an IOI of either 450, 600, or 750 ms. The musical stimuli were two short excerpts (64 notes) of pieces from Bach (“Badinerie”) and Rossini (Ouverture from Wilhelm Tell) presented in a piano timbre at a tempo of 100 beats per minute (IOI = 600 ms). The motor performance was recorded with a MIDI SPD-6 Roland percussion pad. Stimuli were presented in free field via JBL studio monitors (LRS2325p) installed in front of the participant. Metronome sequences were presented first (i.e., in the order of 600, 450, and 750 ms), followed by the musical excerpts. Each trial was preceded by a practice trial. During the whole session, the experimenter was present. In addition, in the experimental groups, spontaneous (i.e., unpaced) tapping was assessed previous to the Experiment by asking the participants to tap as regularly as possible during 1 min at a comfortable, self-chosen pace. The Experiment was run on Max MSP (6.0) software and lasted 15 to 20 min.

### Data Analysis

Prior to the main analyses, raw tapping time series were pre-processed. The taps corresponding to the first 10 tones/beats were removed. Moreover, taps occurring at an inter-tap-interval (ITI) which departed by more than the 3^∗^inter-quartile range from the median ITI in the trial (i.e., outliers) were removed. In addition, a constant MIDI delay of 211 ms was subtracted from tapping data. Sequences of tapping times, relative to the pacing stimuli, were submitted to analyses using circular statistics which represent a valuable option for processing synchronization data (Kirschner and Tomasello, 2009; Pecenka and Keller, 2011; Sowiński and Dalla Bella, 2013). The advantage of circular statistics is that they do not require a one-to-one correspondence between taps and pacing stimuli, a condition rarely met in poor synchronizers (e.g., Kirschner and Tomasello, 2009; Sowiński and Dalla Bella, 2013). In addition, metrics derived from circular statistics have proven to be very sensitive to uncover individual differences in sensorimotor synchronization tasks (Sowiński and Dalla Bella, 2013).

Taps in a sequence were represented on a circle. One full circle (i.e., a 360° scale) indicates the IOI between the periodically recurring pacing events (metronome tones or musical beats). The time of the pacing event corresponds to 0°. Each tap is represented by an angle relative to the time of the pacing event. For example, a tap occurring 150 ms after one of the tones of the metronome sequences with an IOI of 600 ms corresponds to an angle of 90° on the circle. In contrast, a tap preceding the tone by 150 ms is indicated by –90°. For each tapping sequence, the angles corresponding to the taps were transformed into unit vectors, and the mean resultant vector *R* (see Fisher, 1993; Mardia and Jupp, 2000; Berens, 2009) was computed. The vector *R* served to calculate two measures of synchronization abilities, namely *consistency* and *accuracy* (Sowiński and Dalla Bella, 2013). Consistency refers to the variability of the discrepancy between the time of the taps and of the pacing events. High consistency is achieved if the person taps always at the same point in time in relation to the pacing event (e.g., tone or musical beat). Consistency corresponds to the length of vector *R*, with values ranging between 0 and 1. A value of 0 reflects a random distribution of angles around the circle (i.e., at-chance performance), whereas a value of 1 refers to maximum consistency (absence of variability). Accuracy indicates the average difference between the timing of the taps and the timing of the pacing events. Perfect accuracy is achieved if the participant taps exactly at the time of the pacing event. Accuracy is expressed by the angle of the vector *R* (*𝜃* or relative phase, in degrees). It indicates whether participants tapped before (negative angle) or after (positive angle) the pacing event and how close the taps occurred on average to the time of the pacing event. Accuracy values were only calculated if participants’ synchronization performance was above chance, as assessed with the Rayleigh test for circular uniformity (Wilkie, 1983; Fisher, 1993). Rejection of the null hypothesis (i.e., random distribution of data points around the circle) occurs when vector length is big enough (i.e., when taps occurred reliably at a given phase relationship relative to the pacing stimulus). In addition, before performing further analyses, vector length values were submitted to a logit transformation to reduce data skewness, which is typical of synchronization data (e.g., Kirschner and Tomasello, 2009; Sowiński and Dalla Bella, 2013). Finally, in the spontaneous tapping task, the mean ITI was calculated as a measure of preferential tapping tempo and the Coefficient of Variation of the ITIs (CV ITI) was computed as a measure of motor variability.

## Results

**FIGURE 1:**
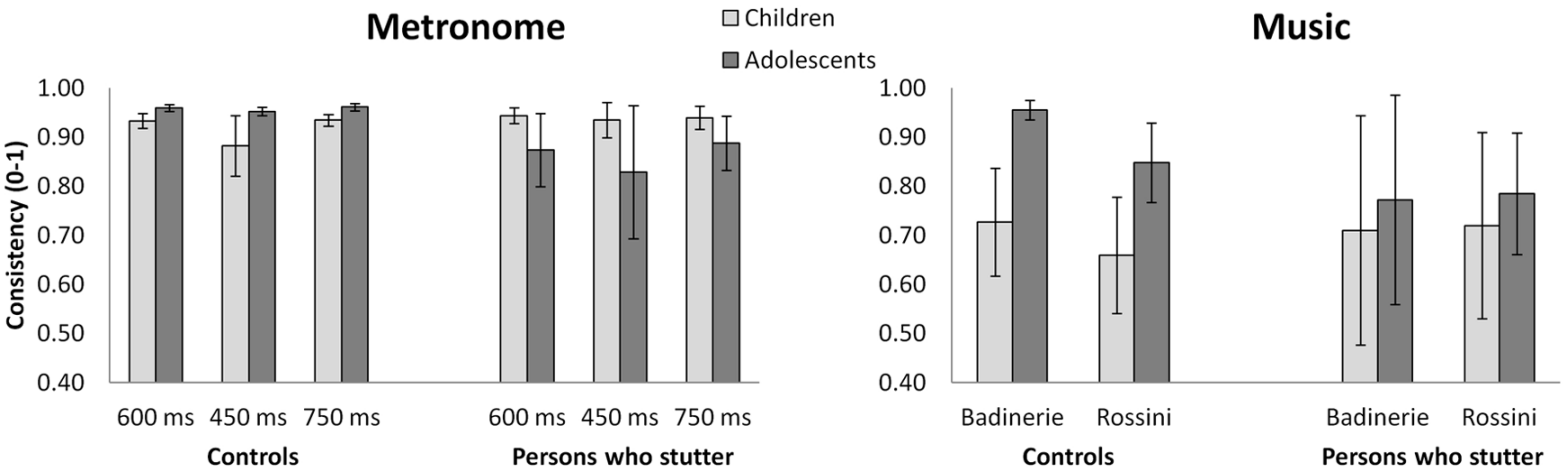
**Mean synchronization consistency for metronome and musical stimuli in both age groups of participants who stutter vs. the control group.** Raw values are shown. Error bars represent 95% confidence intervals.

**FIGURE 2:**
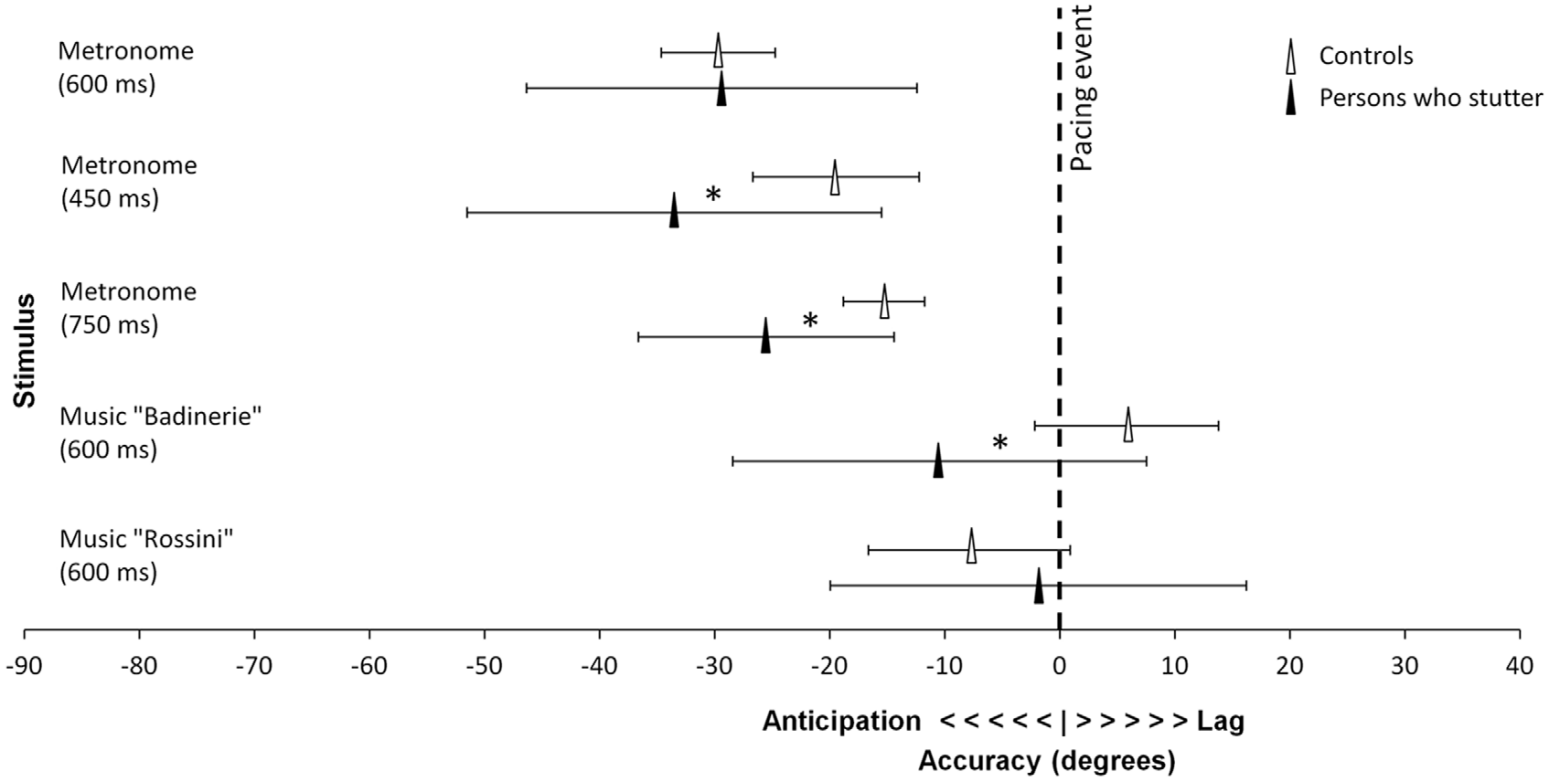
**Accuracy differences between the control group and participants who stutter.** Mean vector angle is displayed averaged over age groups. Significant differences are marked by stars (marginal for 450 ms). Error bars represent 95% confidence intervals.

Mean consistency and accuracy for participants who stutter and controls are presented in **Figures 1** and **2**, respectively. Consistency data were entered in a 2 × 2 × 3 Analysis of Variance (ANOVA). Group (participants who stutter vs. controls) and Age (children vs. adolescents) were both between-subject factors, whereas IOI (450 ms vs. 600 ms vs. 750 ms) was the within-subject factor^2^. Age differences in consistency varied among the groups, as shown by a significant Group × Age interaction [*F*(1,58) = 17.96, *p* = 0.0001, $\eta_{p}^{2}$ = 0.236]. Analysis of simple effects revealed an increase of consistency with age in controls [*F*(1,59) = 7.8, *p* = 0.007, $\eta_{p}^{2}$ = 0.116], whereas participants who stutter showed the reverse pattern [*F*(1,59) = 9.1, *p* = 0.004, $\eta_{p}^{2}$ = 0.134]. Moreover, a tendency for all participants to show lower consistency at the fast tempo (IOI = 450 ms) was observed, but it just failed to reach significance [*F*(2,116) = 2.8, ε = 0.89 *p* = 0.07]^3^. The other interactions were not significant.

To test whether predictive timing differed between the participants who stutter and controls, accuracy data were submitted to 2 × 2 factorial ANOVAs for circular statistics (Harrison–Kanji Test), using the Matlab Circstat toolbox (Berens, 2009), one for each IOI^4^, taking Group and Age as factors. At the slowest tempo (IOI = 750 ms) and at the fastest tempo (IOI = 450 ms), children and adolescents who stutter tapped more in advance of the pacing event than controls did [750 ms, *F*(1,59) = 5.67, *p* = 0.02; 450 ms, *F*(1,59) = 3.82, *p* = 0.06, marginally significant; 600 n.s., *p* = 0.31]. No age differences (*p* > 0.25) and no interaction were found (*p* > 0.45).

The same analyses as above were carried out for synchronization with music^5^. In both the experimental and control groups, adolescents showed greater synchronization consistency than children, as attested by a main effect of Age [*F*(1,58) = 4.19, *p* = 0.04, $\eta_{p}^{2}$ = 0.067]. Furthermore, there were differences between the two excerpts. One excerpt (“Badinerie”) yielded higher consistency than the other stimulus [“Rossini”, *F*(1,58) = 4.48, *p* = 0.04, $\eta_{p}^{2}$ = 0.072]. The Group × Age interaction failed to reach significance [*F*(1,58) = 2.99, *p* = 0.09]. The analysis of accuracy revealed main effects of Group and Age for “Badinerie” only (the interaction being non-significant, *p* > 0.45). With this stimulus, participants who stutter tapped in advance of the beat while controls’ taps lagged after the beat [*F*(1,53) = 4.45, *p* = 0.04]. Additionally, regardless of stuttering, children tapped later than adolescents [*F*(1,53) = 4.09, *p* = 0.05].

In sum, participants who stutter differed in predictive timing from age-matched peers as they anticipated the pacing event more than controls did when they synchronized with a metronome and with more complex musical excerpts. Differences depending on stimulus type were also found for consistency. With music, both groups of participants showed higher consistency with age. However, with metronome sequences, adolescents who stutter were less consistent than children, whereas in the control group consistency increased with age. To examine whether these age differences in the stuttering group could be related to differences in stuttering severity, a correlation between stuttering severity (measured by the SSI-3 scores) and age was computed. Older participants exhibited greater stuttering severity than younger participants (Pearson, two-tailed, *r* = 0.55, *p* = 0.01). Hence, the observation that consistency decreased with age in participants who stutter may have been confounded with differences between the two age groups in terms of stuttering severity.

For this reason we further focused on the relation between stuttering severity and synchronization abilities across the two age groups. Cases of poor synchronization were first identified among individuals who stutter relative to their respective age group. Cutoff scores for consistency and accuracy in each age group were defined for synchronization with each of the five pacing stimuli, based on the performance of the control groups and using *t*-statistics for single-case designs (Crawford and Howell, 1998; Crawford and Garthwaite, 2002). Cutoff scores indicate values for consistency and accuracy at which 2.5% of the estimated population, based on performance of the control groups, fall below the score (see **Table 2**).

**Table 2:** Cutoff-scores for synchronization performance.

Stimulus	Consistency (0–1)		Accuracy (degrees)	
	Children	Adolescents	Children	Adolescents
Metronome 600 ms	0.84	0.91	<–64.26	<–64.68
Metronome 450 ms	0.57	0.89	<–68.36	<–69.34
Metronome 750 ms	0.85	0.91	<–40.88	<–38.84
Badinerie	0.12	0.86	<–33.38	<–44.19
Rossini	0.03	0.34	<–59.33	<–70.73

*For accuracy, only the relevant lower cutoff scores are displayed.*

Thirteen out of 20 participants who stutter exhibited poor synchronization on one or both measures of synchronization with at least one of the stimulus types. Five participants showed low consistency, five low accuracy, and three were affected on both measures relative to controls. Four out of 10 children and 9 out of 10 adolescents showed impaired synchronization.

**FIGURE 3:**
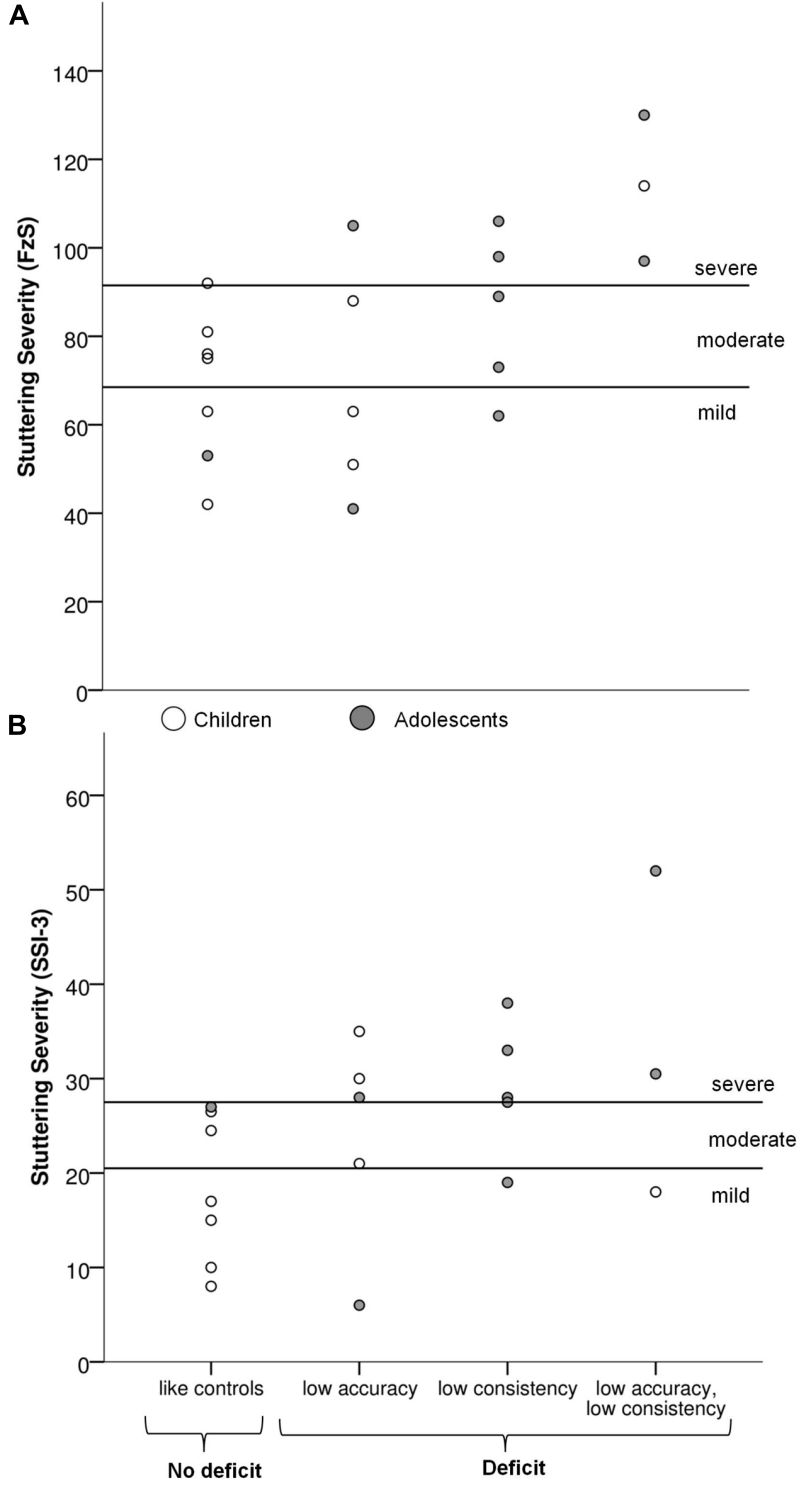
**Individual profiles for participants who stutter depending on their performance in the synchronization tasks plotted against stuttering severity. (A)** Psychosocial severity of stuttering (FzS) **(B)** Severity of stuttering symptoms (Stuttering Severity Instrument, SSI-3).

The link between these individual profiles and stuttering severity was further examined, as illustrated in **Figure 3**. Participants showing low consistency relative to controls were grouped (*n* = 8) and compared to the participants who did not differ from controls (*n* = 12). Participants with low consistency presented more severe stuttering symptoms in both SSI- and FzS-measures^6^ than the participants who exhibited consistency within the range of controls (with SSI, *t*(18) = 2.2, *p* = 0.04, *r*^2^= 0.21; with FzS, *t*(18) = 2.8, *p* = 0.01, *r*^2^= 0.30). The same analysis was performed on accuracy. No differences in stuttering severity were found between participants who stutter showing low accuracy and those participants who were comparable to the control group. In sum, only consistency in a non-verbal synchronization task was associated with stuttering severity, not accuracy.

Finally, additional analyses were conducted in the group of participants who stutter to test whether their poor timing performance was specific to synchronization or whether it more generally concerned the production of a rhythmic sequence, even in the absence of a pacing stimulus (i.e., in the unpaced tapping task; for data on children without disorders, see Drake et al., 2000; McAuley et al., 2006). No significant correlation was found between consistency obtained in the paced tapping tasks and variability in unpaced tapping (i.e., CV ITI). Moreover, participants who stutter showing low synchronization consistency did not differ in motor variability from participants without synchronization deficits (mean CV ITI = 0.07, *SD* = 0.03 in both sub-groups).

## Discussion

In the present study, we examined non-verbal timing abilities in children and adolescents who stutter, with a focus on predictive timing, tested with a sensorimotor synchronization task. Participants who stutter showed poorer synchronization to a rhythmic auditory stimulus such as a metronome or music (i.e., lower accuracy or consistency) than age-matched peers. Examining individual synchronization profiles revealed that 65% of participants who stutter displayed timing deficits. Adolescents who stutter were more impaired (in particular in consistency) than children. Low synchronization accuracy was reflected by the fact that participants who stutter tapped earlier in relation to the pacing stimulus as compared to controls. Low synchronization consistency (i.e., higher variability) was observed in particular in participants with severe stuttering. These differences in synchronization performance associated with stuttering severity were unlikely to result merely from general motor impairment, as indicated by the performance in an unpaced tapping task.

These results provide for the first time evidence of a very specific deficit in synchronization accuracy in children and adolescents who stutter, namely a consistent bias toward over-anticipating the moment of occurrence of the pacing event. This is manifest in their tendency to tap earlier in relation to the auditory pacing stimulus than controls (i.e., they showed a higher negative mean asynchrony – NMA – than controls). This finding is in keeping with models of stuttering underscoring the role of predictive timing in the disorder (Harrington, 1988). Furthermore, we provided evidence for a link between lower synchronization consistency and stuttering severity in the tested age groups. Synchronization performance also differed from spontaneous rhythmic activity in the absence of a pacing stimulus (i.e., in unpaced tapping). These results suggest that stuttering may be associated with a general-purpose timing deficit that pertains specifically to sensorimotor tasks requiring auditory–motor integration in children and adolescents. This intriguing hypothesis deserves further enquiry.

Synchronization accuracy and consistency deficits can derive from several and probably independent sources. The fact that individuals who stutter over-anticipated the pacing stimulus (i.e., they showed larger NMA) than controls may shed light on the nature of the underlying malfunctioning timing mechanism. NMA is a common phenomenon in synchronized tapping to simple periodic stimuli such as a metronome (see Aschersleben, 2002; Repp, 2005; Repp and Su, 2013). With more complex stimuli, such as music, NMA is typically smaller or may disappear. NMA is likely to be linked to perceived synchronicity and predictive timing, that is, the estimation of the time when a self-produced motor response and an external periodic auditory event will coincide (Białuńska et al., 2011; Repp and Su, 2013). In this respect, the higher NMA would act as a non-verbal indicator of a predictive motor timing error.

In the timing literature, several accounts of the origins of the NMA have been proposed. The sensory accumulation hypothesis (Aschersleben, 2002) proposes that proprioceptive tactile feedback is integrated more slowly than auditory information by the central nervous system. As a consequence, taps are produced in advance in order to subjectively coincide with the percept of the auditory stimulus. In this perspective, the higher NMA in stuttering participants may derive from a slower or less efficient kinesthetic integration function. Previous studies on manipulation of kinesthetic feedback of orofacial effectors pointed in this direction (e.g., Archibald and De Nil, 1999; Loucks and De Nil, 2006; Namasivayam et al., 2009). For example, adults who stutter show reduced acuity of jaw movements when depending on kinesthetic feedback alone (Loucks and De Nil, 2006). Increasing orofacial kinesthetic feedback aids persons who stutter in stabilizing their speech production at fast speech rates (Namasivayam et al., 2009). Alternatively, a purely perceptual account of the NMA proposes that it is due to temporal underestimation of the IOI (Wohlschläger and Koch, 2000). In keeping with this account, participants who stutter may have underestimated the duration of the IOI more than controls did. To date, there are very few studies that tested temporal perception and estimation in stuttering. In a recent experiment on the perception of millisecond differences in voice onset times, Neef et al. (2012) observed that adults who stutter show a larger range of ambiguous perceptions between voiced and voiceless German plosives than adults who do not stutter. In temporal estimation paradigms for larger time lapses (i.e., 30 s), adults who stutter showed less accurate estimation, in particular those participants that were severely stuttering (Ezrati-Vinacour and Levin, 2001). In a very recent study, Wieland et al. (2015) showed that children who stutter aged 6 to 11 years have greater difficulty than controls in discriminating rhythmic sequences of tones. This finding points toward a deficit in rhythm perception that could also relate to impaired rhythm production. Further studies should shed light on the role of perceptual and proprioceptive auditory–motor mechanisms in motor and speech synchronization tasks with participants who stutter.

Consistency deficits can also be linked to a faulty auditory–motor coupling mechanism. Although there are various sources of variability in periodic motor tasks, such as central and peripheral mechanisms (i.e., central timekeeping vs. motor effector variability, see Wing and Kristofferson, 1973), adaptation to the auditory stimulus requires both phase and period correction, which are critical processes to maintain synchronization in paced tapping tasks (see Repp, 2005, 2006). It is worth noting that some previous studies also reported higher motor variability in unpaced motor tasks (i.e., reproducing the same time interval without a pacing stimulus) in stuttering. In the two studies focusing on timing abilities in children who stutter, higher motor timing variability in the unpaced continuation phase of a synchronization–continuation task was observed (Howell et al., 1997; Olander et al., 2010). In adults who stutter, evidence was at times pointing toward higher (Subramanian and Yairi, 2006) or lower motor timing variability (Brown et al., 1990) during unpaced tapping relative to controls. Based on our findings, we cannot exclude generally increased motor variability in stuttering. Nevertheless, because the difficulties in coupling movement to a predictable auditory stimulus were unrelated to motor variability in unpaced tapping, our findings suggest that coupling of perception and action may represent an additional source of impairment, linked to stuttering severity.

Deficient processes relevant to auditory–motor integration have been discussed in models of stuttering. Neilson and Neilson (1987) were among the first to propose that a basic problem in stuttering is the formation or access of auditory–motor models in speech processing. They based their proposal on tracking experiments with visual and auditory stimuli that showed that adults who stutter performed significantly poorer than a control group with auditory stimuli than with visual stimuli. Recent models also assume that stuttering is based on sensorimotor predictions that are not correctly integrated with sensory feedback information during speech production (Max et al., 2004; Civier et al., 2010; Hickok et al., 2011). In terms of temporal processing, we can speculate about several mechanisms which can be the locus of impairment in stuttering. First, there may be a lack of precise temporal predictions generated by unstable or deficient forward-models (compatible with Max et al., 2004), whether in fine motor or in speech production. Alternatively, predictions may be subject to a temporal delay and therefore, may not appropriately match with feedback information (Harrington, 1988). Finally, temporal predictions and forward-models may be correct, but a temporal mismatch may still occur because sensory feedback is too slow or not aligned in time with the predictions (compatible with Civier et al., 2010).

Another question is to what extent the relatively long non-speech movements tested in our tasks can inform about temporal prediction mechanisms directing speech movements on a much smaller time scale. The frequencies of speech movements are among the highest in human behavior, ranging from 9.5 to 21 phones/second or from 1.5 to 6 syllables/second (e.g., Koreman, 2006; de Jong and Wempe, 2009). In the present and in previous synchronization studies with persons who stutter, time intervals were tested at lower movement rates adequate for manual synchronization, often not yielding significant differences (70–300 beats/minute; Hulstijn et al., 1992; Zelaznik et al., 1994; Max and Yudman, 2003; Neef et al., 2011; see also Repp, 2003, 2006). Slower movement rates may put less demand on timing mechanisms than speech does and thereby may fail to show differences in predictive timing between persons who do and do not stutter. However, this does not preclude the possibility of temporal prediction deficits at faster rates. This possibility should be carefully assessed in further studies with adults and children using other appropriate tests (e.g., verbal tasks; perceptual timing tasks).

For children and adolescents who are still developing timing abilities in both speech and non-verbal behavior (Drake et al., 2000; McAuley et al., 2006), task demands during synchronization seemed to be adequately high to reveal group differences in our study. However, an analysis of individual differences indicate that synchronization tasks were sensitive in particular to severe timing deficits. Synchronization deficits were visible in all eight participants who stutter with severe SSI-3. In the group of participants with mild to moderate stuttering, 40% showed differences compared to their age-matched peers. Remarkably, in our sample of participants who stutter, consistency and accuracy deficits did rarely co-occur. Only three participants (15%) were impaired at both levels. A subgroup (25%) displayed particularly low consistency, but accuracy was comparable to controls. Another subgroup (25%), in contrast, displayed low accuracy (i.e., high NMA), but consistency was as high as in controls. In this subgroup, one of the participants exhibited synchronization consistency which was even superior to that of the control group (mean vector length = 0.99, *SD =* 0.005). Interestingly, one of the previous studies also reported less variable motor performance in adults who stutter than in controls during unpaced finger tapping and oromotor movement tasks (Brown et al., 1990). The authors suggested that excessive consistency could reflect a less flexible and adaptive motor system. Although a larger group of participants is needed to confirm a bimodal distribution, these results point to subgroups which could be indicative of different timing mechanisms or compensatory strategies underlying stuttering.

The observed link between stuttering severity and consistency deficits in synchronization opens interesting perspectives for future studies. Severe stuttering as measured by the SSI at age 8 is the most reliable predictor of persistent stuttering (Howell and Davis, 2011). Eighty percent of the children showing stuttering symptoms develop a merely temporary disorder which will disappear by adulthood (Yairi and Ambrose, 1999). In the remaining 20%, however, stuttering does persist into adulthood. From age 16 on, recovery from stuttering is highly unlikely. Recovery rates before 16 years are around 50% for children aged 8 years, and around 25% when aged 10 years; recovery above 12 years is only occasionally reported (Andrews and Harris, 1964; Howell and Davis, 2011; for a review, see Howell, 2011). In our study, synchronization deficits were most visible in the group of severely stuttering participants which were also more numerous among the adolescents who were more prone to persistent stuttering than children. If the likelihood of persistence of stuttering is related to the severity of an underlying timing deficit, non-verbal synchronization ability could be an indicator of the persistence of stuttering. Moreover, given the results of Chang and Zhu (2013) on attenuated connectivity in neural timing circuits, it would be valuable to test younger children near the age of stuttering onset on their non-verbal synchronization abilities, stuttering symptoms and corresponding neural correlates in verbal tasks. Another aspect worth considering is that persons who stutter exhibit deficits in learning motor sequences (Smits-Bandstra et al., 2006; Smits-Bandstra and De Nil, 2007, 2009, 2013). It is unclear whether these deficits are associated or co-develop with deficits in coupling action and perception (e.g., sensorimotor synchronization) during childhood and adolescence – a possibility for future investigation. Finally, as other developmental speech disorders have recently been proposed to jointly emerge with deficits in the non-verbal domain (e.g., reading disabilities, Woodruff Carr et al., 2014), it will be relevant to address the specificity of timing deficits in stuttering, as indicated by our results, in comparison with other disorders in future research.

To conclude, children and adolescents who stutter in our sample showed deficits in sensorimotor synchronization compared to age-matched peers. Deficits were visible in synchronization accuracy, as well as in consistency, but affected different subgroups of participants. In addition, synchronization consistency was indicative of stuttering severity, a relation which could be of potential interest for future studies. Accuracy deficits pointed to altered mapping of perception to action and were indicative of impaired predictive timing. In sum, our results lend support to the hypothesis that stuttering is associated with timing deficits in young age and are found in the non-verbal domain. Whether these deficits are pertaining more to perception, sensorimotor integration or motor planning and how they reflect verbal timing has to be clarified in future research.

## Conflict of Interest Statement

The Guest Associate Editor Lauren Stewart declares that, despite having collaborated with author Simone Dalla Bella, the review process was handled objectively. The authors declare that the research was conducted in the absence of any commercial or financial relationships that could be construed as a potential conflict of interest.

## Footnotes

1 See recent findings presented by the same research group (Hilger, Zelaznik, and Smith) at the 2014 Convention of the American Speech-Language-Hearing Association (ASHA).

2 Data for one participant from the control group could not be included because of failure to complete the 750-ms condition.

3 The Greenhouse–Geisser correction for inhomogeneity of variance was applied when appropriate. Uncorrected degrees of freedom, epsilon, and probability level following correction are reported.

4 To our knowledge, there is no 3-way circular ANOVA. For that reason three 2-way ANOVAs were computed.

5 Consistency data was excluded for one participant of the test group who was not able to synchronize with one musical excerpt (Rossini). Accuracy data of nine participants was not entered in the analyses as the Raleigh test did not reach significance (i.e., participants’ performance was at chance).

6 Note that SSI and FzS-Scores were not correlated in our participant group.
